# Women in Translational Medicine: Tools to Break the Glass Ceiling

**DOI:** 10.3389/fmed.2018.00330

**Published:** 2018-12-13

**Authors:** Sophie H. Bots, Mira G. P. Zuidgeest, Aisha Gohar, Anouk L. M. Eikendal, Alessandra Petrelli, Harmieke van Os-Medendorp, Marieke F. van der Schaaf, Nina M. van Sorge, Myriam van Wijk, Sabine Middendorp, Caroline M. Speksnijder, Kerstin Klipstein-Grobusch, Vicky Seyfert-Margolis, Esther Mollema, Femke van Wijk, Hester M. den Ruijter

**Affiliations:** ^1^Laboratory for Experimental Cardiology, University Medical Center Utrecht, Utrecht University, Utrecht, Netherlands; ^2^Julius Global Health, Julius Center for Health Sciences and Primary Care, University Medical Center Utrecht, Utrecht University, Utrecht, Netherlands; ^3^Julius Center for Health Sciences and Primary Care, University Medical Center Utrecht, Utrecht University, Utrecht, Netherlands; ^4^Diabetes Research Institute IRCCS San Rafaele Scientific Institute, Milan, Italy; ^5^Department of Dermatology and Allergology, University Medical Center Utrecht, Utrecht University, Utrecht, Netherlands; ^6^Center for Research and Development of Education, University Medical Center Utrecht, Utrecht University, Utrecht, Netherlands; ^7^Medical Microbiology, University Medical Center Utrecht, Utrecht University, Utrecht, Netherlands; ^8^University Medical Center Utrecht, Utrecht University, Utrecht, Netherlands; ^9^Pediatric Gastroenterology, Wilhelmina Children's Hospital and Regenerative Medicine Center, University Medical Center Utrecht, Utrecht University, Utrecht, Netherlands; ^10^Department of Oral and Maxillofacial Surgery and Special Dental Care, University Medical Center Utrecht, Utrecht University, Utrecht, Netherlands; ^11^Department of Head and Neck Surgical Oncology, University Medical Center Utrecht Cancer Center, Utrecht University, Utrecht, Netherlands; ^12^Department of Epidemiology and Biostatistics, School of Public Health, Faculty of Health Sciences, University of the Witwatersrand, Johannesburg, South Africa; ^13^MyOwnMed, Bethesda, MD, United States; ^14^HPO Center, Hilversum, Netherlands; ^15^Laboratory of Translational Immunology, University Medical Center Utrecht, Utrecht University, Utrecht, Netherlands

**Keywords:** glass ceiling, gender, translational medicine, gender roles, gender champions

## Abstract

Despite the recent movements for female equality and empowerment, few women occupy top positions in scientific decision-making. The challenges women face during their career may arise from societal biases and the current scientific culture. We discuss the effect of such biases at three different levels of the career and provide suggestions to tackle them. At the societal level, gender roles can create a negative feedback loop in which women are discouraged from attaining top positions and men are discouraged from choosing a home-centred lifestyle. This loop can be broken early in life by providing children with female role models that have a work-centred life and opening up the discussion about gender roles at a young age. At the level of hiring, unconscious biases can lead to a preference for male candidates. The introduction of (unbiased) artificial intelligence algorithms and gender champions in the hiring process may restore the balance and give men and women an equal chance. At the level of coaching and evaluation, barriers that women face should be addressed on a personal level through the introduction of coaching and mentoring programmes. In addition, women may play a pivotal role in shifting the perception of scientific success away from bibliometric outcomes only towards a more diverse assessment of quality and societal relevance. Taken together, these suggestions may break the glass ceiling in the scientific world for women; create more gender diversity at the top and improve translational science in medicine.

## Introduction

Translational medicine is a rapidly growing field of scientific research. For this research discipline to be successful, a multi-disciplinary, and highly collaborative approach is required. Both men and women are needed to contribute to this important field of research. However, women and men are different. Not only in terms of the biology of sex hormones and sex chromosomes but also in terms of gender roles in society. Acknowledging that these differences matter has brought about an inspirational movement of sex and gender integration in biomedical research (1). Funding agencies and journals now guide and instruct authors to include sex and gender in their analyses to improve biomedical research and healthcare provision. In addition, gender diversity in leadership has become a serious target for many organisations, not in the least because there is a growing body of evidence that gender diversity in executive teams positively correlates with (financial) performance^1^ and that gender-diverse teams produce better quality science (2).

Despite the recent movements for female equality and empowerment, it is clear that gender bias still exists. A Dutch study (3) shows that while younger women (aged < 45) are more highly educated than men in the same age category, they seem to be unable to translate their educational advantage into better career chances. The 2018 Global Education Monitoring Report showed that women were underrepresented in university leadership positions across the globe (4). Particularly in science, despite fairly balanced ratios of male-to-female undergraduates and post-graduates, women are less likely to progress through the career ladder than men, resulting in a low representation of women in senior positions. For instance, a report from the Association of the American Medical Colleges (5) indicates unequal distribution of chairs by gender basis, with a total of 15.8% women in Academic Medicine in 2015.

So what are the obstacles that prevent so many female scientists from occupying top positions in scientific decision-making? How can we explain the steep fall in percentage of women with each step up on the career ladder (6)? Finally, which structural interventions can be implemented at the institutional level to promote women's careers in science? We believe there is a need for a multilevel approach consisting of a combination of bold methods that address the deeply rooted causes that lead to gender disparity in the selection procedures for professorships and the most senior positions within companies. In this article, we discuss possible reasons for gender disparity at three levels or stages in the career. The first and broadest is the societal level, which influences the career of women throughout their lives. The second and more specific level covers bias in hiring practices, which is most important at the start of one's career. The third discusses the effect coaching and evaluation may have during the career. We will also propose suggestions that may tackle some of the inherent biases present in each stage.

## The Societal Level: The Effect of Gender Roles

The hampered progression of women in science is known as the “glass ceiling,” which is the resistance women (and minorities) face when they attempt to reach the top ranks of management in organisations. One of the deeply rooted causes for this glass ceiling may be the societal role of women, which dictates gender-specific and accepted behavioural patterns. Men are naturally expected to be the main provider for the family, whereas women are expected to take care of the family and household. These societal expectations are reflected in the work environment. The current organisation culture values masculine traits and is therefore more attractive for and more facilitating towards men. One intrinsic hurdle of this culture for women is the effect pregnancy has on the progression of their careers. Women who either are pregnant, are planning to get pregnant or recently had a child often face negative consequences in their careers such as the termination of their contract or being denied a promotion because of the implicit expectation that they will need to take time off and reduce their work effort due to their maternal duties. This happens to 43% of women in the Netherlands (3). Another example comes from Japan, where one university deliberately excluded female applicants from medical school because they were expected to take time off during their studies for family-related duties.^2^

These kind of intrinsic mechanisms and other parts of the glass ceiling feed into a downward spiral. Women make different choices during their career based on societal and work-related expectations and often end up with more limited choices in the end compared to men. One example of this is that senior positions are made available during the years in which women tend to have children and are thus likely to be given to their male counterparts. However, on the other hand, when a woman goes against societal expectations of maternal duties, they receive stigma and criticism from society. This makes it difficult to break the vicious cycle and leads to women preferring jobs that enable a good work-life balance. These preferences in turn lead to crowding, in which female-dominated jobs are valued less compared to male-dominated jobs demonstrated by lower salaries, few stable long-term contracts, and an abundance of part-time jobs (7).

This feedback loop may start already early in life. According to the *preference theory* (8), women make their choice between family and business based on their preference for a particular lifestyle: work-centred, home-centred, or adaptive (combining paid work and family time). Women might adjust their preferences as a response to gender inequality, adapting to the current social disparities and expectations. These preferences feed into the vicious cycle described above and are formed early on in life. This makes it difficult to later redistribute roles and responsibilities more equally between men and women, which ultimately negatively impacts women's career prospects and possibly their mental health (9). The same is true for men who go against societal expectations by adopting a home-centred lifestyle instead of a work-centred one.

Therefore, we call for a societal change on the views of gender roles. The double-duty that women often do in terms of unpaid domestic labour and progression of scientific careers highlights their capability and creativity, which should be valued by our society. Female translational scientists should be aware of behavioural differences between men and women and should use this knowledge to adapt accordingly. While masculinising their behaviour can help to be taken more seriously, it can also have negative effects on how women are perceived socially. Both women themselves and society should thus value female-specific behavioural traits and use these to their advantage.

Changing the societal role of women is an ongoing process and will take time and effort to be accomplished. A gender-balanced educational workforce at different educational stages, from school to university and workplace, may help the progression of women's self-awareness and careers. Schools can play an important role in breaking the vicious cycle early on. Teachers should be made aware of unconscious biases present in their teaching material and update them accordingly (10). Schools can invite female scientists to talk about their work and act as role models for young girls who aspire a career in science (11). Mainstream media is also an important source of inspiration and empowerment for young and adolescent girls. The introduction of strong female superheroes such as *Wonder Woman* provides girls with role models that break traditional gender roles (12). Opening up the discussion about gender roles at a younger age and providing girls with enough female role models may empower them to challenge and go beyond societal expectations.

## Hiring Practices: Looking Beyond Gender Bias

People make decisions that are often incorrect and not based on facts, even though we sincerely think that we objectively made the best choice. Deep-rooted prejudices around male leadership and the belief that men are better at math and science continue to influence hiring practices (13, 14). These ideas are perpetuated by key public figures in science such as a former Harvard President (in 2005) and the former President of the Royal Academy of Sciences of the Netherlands (in 2018). They attribute the underrepresentation of women in science and scientific institutions to “issues of intrinsic aptitude” and “lack of willingness to put in the required hard work which is needed for scientific excellence.”

The first step towards dealing with heuristics and biases is to acknowledge they exist and understand how they work. The next step is to overcome them, for example through changing current selection procedures. We highlight three possible measures that can be used to create more female-friendly selection procedures in scientific institutes.

### Using Artificial Intelligence to Pre-Select Suitable Candidates

Organisations outside of the scientific world have already experimented with new recruitment approaches that might improve the gender balance of selected candidates. One example of this is Unilever, one of the world's leading consumer-goods conglomerates with 170,000 employees worldwide. They integrated machine learning approaches in their talent recruitment programme, using neuroscience-based games, and LinkedIn profile information to determine whether a given candidate fits the job requirements^3^. Each candidate had to complete a standardised online interview and their responses were analysed using artificial intelligence. Afterwards, the hiring managers were given a detailed list of candidates the programme deemed most suitable for the position. By using such algorithms to aid the selection process, Unilever hired their most diverse class to date not only regarding gender, but also ethnicity and socioeconomic class^2^. Adopting this type of algorithm-based pre-selection system would allow scientific institutions and universities to streamline their hiring processes in an unbiased manner. Because human judgment still plays an important role in the final decision to hire a candidate, it is also important to educate recruiters and human resources staff on how to retain diversity during the hiring process. Both adding technology to the screening process and increasing awareness under recruiting staff about gender biases may help to make hiring practices more gender-balanced.

### Training of Selection Committees Through Gender Champions

Selection criteria for job candidates and decisions made during the selection process lack transparency and are too often made by male-dominated committees with an explicit preference for men (15). Interestingly, women in leading positions of masculine organisations more often choose a male candidate over a female one because they have internalised the masculine behaviour of their peers (the “Queen Bee” effect). In contrast, women in leading positions of more gender-balanced organisations are more open to mentorship and sponsorship of other women (16). Because both men and women are biased towards male candidates in a male-dominated atmosphere, adding more women to the selection committee may even out the playing field. A successful example of this approach comes from intervention studies in hiring committees to select young faculty (17).

Inspired by the integration of gender in biomedical research, we propose to implement institutional gender champions (18). These gender champions, defined as decision-makers with expertise regarding the role of gender in hiring practices, will be included in selection panels to point out any biases in the panel's decision making. In addition, selection panel members will be trained in various aspects that help increase bias awareness, including items such as tests to gain insight in personal unconscious biases^4^, serious games to highlight common interview situations and interpretations, and showcasing the success of female professors. Decision support tools can also support committees by making selection criteria more objective and reach a more structured and transparent decision based on facts instead of feelings.

## Coaching and Evaluation: Towards a Level Playing Field

Low self-confidence and self-perception among women may be another cause of gender disparity. Girls from six years of age are already less likely to perceive themselves as brilliant than boys of the same age (19). Small and unintended implicit suggestions on male superiority in our society may engrain the idea that men and boys are superior in leadership positions from a very young age. This is perfectly illustrated by the recently withdrawn girls shoe line by Clarks^Ⓡ^ called “Dolly Babe,” for which the equivalent version for boys (which is still available) was called “Leader.” Over time, women may internalise the feelings of professional inferiority that are implicitly suggested by such incidents and grow to believe themselves underequipped for their job or academic studies. This is also known as the *imposter syndrome* (20). Early research into this condition labeled it as a female condition; however, although men are less likely to report it due to stigma, more recent research has shown that they struggle with imposter syndrome as well (21). People suffering from imposter syndrome may not pursue the career they wish to have due to their feelings of inaptitude, instead settling for less. Early recognition of the condition and appropriate support can help individuals deal with these feelings and thus help them reach their full potential (21).

### Expanding Coaching and Sponsoring Programmes

We therefore propose to expand coaching and sponsor programmes to better suit the needs of women (and men) aspiring a career in science. In 2012, the University Medical Center Utrecht (The Netherlands) implemented a talent programme to promote scientific careers of women called the Steyn Parvé programme. Five years later, the percentage of female professors had increased from 18 to 26%. A similar trend was seen at Vita-Salute San Raffaele University (Milan, Italy), where the percentage of female professors increased from 12 to 25% in the 10-year period. This increase occurred naturally, without the need for adopting any institutional policy to promote gender equality. The British Medical Association (BMA) in the United Kingdom (UK) organises an annual one-day conference celebrating and promoting women in science^5^. The association also advocates the use of role models so that women early on in their career have an inspirational figure to look up to for direction and for examples of what can be and how it can be achieved. Recent data from the Wellcome Genome Campus in the UK show that the implementation of an integrated “Sex in Science” programme, including mentoring and addressing unconscious biases in hiring practices, helped increase the percentage of female employees overall and the percentage of female speakers at seminars and conferences (22). Introduction of a mentoring programme at the Flinders University in Australia markedly improved both the success and the self-esteem of junior academic women (23). The extension of mentoring programmes may be one of the key determinants of academic success in medicine (24), thus good mentoring and having female role models may encourage women to proceed in science. We believe that the development of professional mentoring skills should be implemented as early as possible in career tracks. Mentoring programmes or workshops should address amongst other qualities of a good mentor, mutual responsibilities, giving feedback, bias and diversity, and mentorship pitfalls. The chance that one person can fulfill all mentoring needs in any phase of a career is small and mentorship programmes should also support the development of mentor networks (25). With a mentor network, mentorship can be diverse in age (e.g., peer-mentoring), rank, area of expertise, and gender and may therefore be more effective (25). This is in line with the policy of gender equality promotion supported by the League of European Research University (LERU), a network of research-intensive universities based in Europe. They have recommended measures, such as defining clear selection criteria, educating selection committees on implicit gender bias, and involving external evaluators, which should be implemented in all research institutes (26).

Next to mentors, sponsors may also play an important role in advancing to an academic leadership position. Sponsors have the power and position to advocate for unrecognised talent in discussions on executive leadership positions (27) and can play a crucial role in identification, visibility, and training of female talents. However, women are less likely than men to have a sponsor (28). Therefore, sponsorship of women should be promoted, for example by asking every senior leader to adopt at least one female talent. Mentoring and sponsorship should be complemented by funding. Institutes should receive financial incentives to perform research if gender equality policies are to be effectively implemented. In the UK, for example, the Athena SWAN programme gives out gender equality awards to institutes or departments who commit to advance gender equality for academic staff. To be considered for funding from the National Institute for Health Research, institutes should have at least a silver-level Athena SWAN award. Similar incentive structures could be implemented in research institutes across the world.

### Changing the Measure of Scientific Success

Scientific success is often measured using bibliometrics such as the h-index, although the discontent about these measures is growing in the scientific community because they are heavily dependent on the quantity of output instead of the quality. The focus on quantity puts women at a disadvantage, as they have been shown to on average publish less papers compared to their male counterparts throughout their scientific careers (29). However, the papers women publish seem on average to be of higher quality than those of men, suggesting that the lower productivity of women is not due to lack of aptitude (29). Incorporating such insights into the metrics for scientific success may help to level the playing field for women scientists. This would also fit into the general movement beyond metrics that is currently going on in science, which can be referred to as “Science in Transition” (30).

## Conclusion

**Figure 1:**
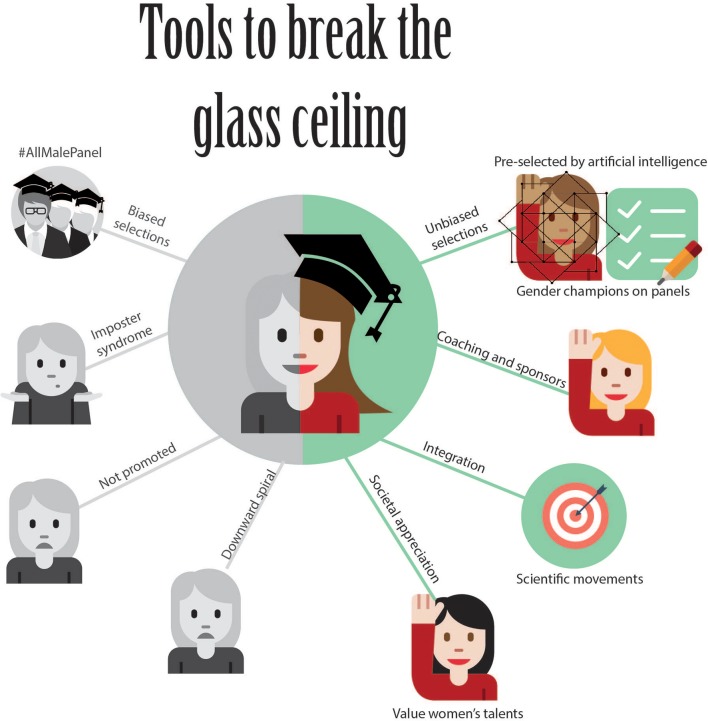
Infographic summarising approaches to break the glass ceiling.

We have discussed several aspects that may prevent female translational scientists from embarking on successful career paths and we have proposed possible solutions to break these barriers (see Figure 1). The approaches described above take gender as the starting point, but are equally applicable to dealing with other disadvantaged minority groups. This application would thus not only improve gender diversity in leadership but diversity in general, increasing the chance of successful translational medicine.

## Author Contributions

All authors listed have contributed to the research underlying and writing of the article. All authors approved the article for publication.

### Conflict of Interest Statement

VS-M was employed by company MyOwnMed, Inc. AP is supported by the Marie Sklodowska-Curie Research Fellowhip Programme. The remaining authors declare that the research was conducted in the absence of any commercial or financial relationships that could be construed as a potential conflict of interest.

## Footnotes

^1^https://www.mckinsey.com/business-functions/organisation/our-insights/why-diversity-matters

^2^https://www.natureindex.com/news-blog/lost-in-japan-a-generation-of-brilliant-women

^3^https://www.businessinsider.nl/unilever-artificial-intelligence-hiring-process-2017-6/?international=true&r=US

^4^https://mindbugstest.nl/mindbugstest/gender-leiderschap/

^5^https://www.bma.org.uk/collective-voice/committees/medical-academics-committee/women-in-academic-medicine
